# Association Between Periodontal Disease and Cognitive Impairment: A Systematic Review and Meta-Analysis

**DOI:** 10.3390/dj14100678

**Published:** 2026-10-18

**Authors:** Hassan Alsaigh, Mishali Alsharief, Abdulmajeed A. Aljabr, Eman Aljoghaiman, Faisal E. Aljofi, Marwa Madi

**Affiliations:** 1 Fellowship in Periodontics, Department of Preventive Dental Sciences, College of Dentistry, Imam Abdulrahman Bin Faisal University, P.O. Box 1982, Dammam 31441, Saudi Arabia; 2230600019@iau.edu.sa; 2 Department of Preventive Dental Sciences, College of Dentistry, Imam Abdulrahman Bin Faisal University, P.O. Box 1982, Dammam 31441, Saudi Arabia; msalsharief@iau.edu.sa (M.A.); ealjoghaiman@iau.edu.sa (E.A.); fealjofi@iau.edu.sa (F.E.A.); 3 Department of Preventive Dental Sciences, College of Dentistry, Majmaah University, Al Majmaah 11952, Saudi Arabia; a.jabr@mu.edu.sa

**Keywords:** periodontal disease, cognitive impairment, Alzheimer’s disease, systematic review, inflammation, neuroinflammation, periodontal medicine, oral-systemic link, meta-analysis

## Abstract

**Background:** Cognitive impairment is an increasing global health burden, and growing evidence suggests that periodontitis may contribute to cognitive decline through chronic inflammation and other oral-systemic pathways. This systematic review aimed to evaluate the association between periodontal disease and cognitive impairment in adults and to contextualize the findings within prior evidence syntheses. In this review, periodontitis is conceptualized as a host-mediated chronic inflammatory burden rather than as a marker of intraoral infection alone. **Methods:** A PRISMA-based systematic review was conducted using PubMed, Web of Science, and Scopus for observational studies published between 2015 and December 2025. Eligible studies included adults assessed for both periodontal status and cognition using validated clinical, radiographic, and neuropsychological measures. Risk of bias was assessed with the Newcastle–Ottawa Scale, and the certainty of the evidence was judged qualitatively. Database-specific search strategies are provided. **Results:** Nine studies met the inclusion criteria, including prospective cohort, cross-sectional, and Alzheimer disease cohorts. Across studies, more severe periodontitis was consistently associated with poorer cognitive performance, higher odds of mild cognitive impairment, and faster cognitive decline. In the four studies eligible for quantitative synthesis, the random-effects estimate indicated an approximately twofold overall association estimate (2.12; 95% CI 1.51–2.98), which is best interpreted as a summary association measure rather than a precise risk estimate. In Alzheimer disease cohorts, periodontal inflammation was associated with accelerated deterioration, including a six-fold greater rate of cognitive decline in one study and faster worsening on standardized cognitive scales in another. Risk-of-bias assessment showed three studies at low risk and six at moderate risk, with no study judged high risk. However, the overall certainty of the evidence was considered low because the evidence base was observational, clinically heterogeneous, and vulnerable to residual confounding and possible publication bias; this judgment reflects a qualitative interpretation of the included study designs and their methodological limitations rather than a formal GRADE rating. **Conclusions:** The current evidence supports a consistent, approximately twofold overall association between periodontal disease and cognitive impairment, broadly in line with prior systematic reviews and meta-analyses, while adding newer cohort and Alzheimer disease data through 2025. The exposure is best understood as a host-mediated periodontal inflammatory burden, and Periodontitis may represent a potentially modifiable contributor to adverse cognitive outcomes, but causal inference and policy-level screening recommendations require stronger interventional and mechanistic evidence. **Clinical Relevance:** Periodontitis represents a potentially modifiable risk factor for cognitive decline and accelerated deterioration in Alzheimer’s disease patients, highlighting the need for interdisciplinary collaboration between dental and medical professionals. Integrating routine periodontal screenings and proactive oral care into the management of aging populations could serve as a valuable, low-risk strategy to help mitigate adverse cognitive outcomes.

## 1. Introduction

Cognitive impairment represents a major public health challenge with significant societal and individual impacts. As the global population ages, the prevalence of cognitive decline is rising, reducing independence and quality of life while escalating caregiver burden and healthcare costs [1]. Cognitive impairment exists along a clinical spectrum that progresses through distinct stages. The earliest phase, known as the preclinical or latent stage, is characterized by normal performance on neuropsychological tests despite biomarkers or imaging evidence of disease pathology. This may progress to Mild Cognitive Impairment (MCI), a prodromal stage where objective cognitive deficits are measurable but daily functioning remains preserved. The final stage is dementia, the full expression of the disease, characterized by severe impairment that interferes with activities of daily living such as eating, dressing, or grooming.

Emerging research increasingly highlights the “oral-systemic link,” specifically focusing on how chronic oral infections like periodontitis may influence remote organs, including the brain. Periodontal disease is a ubiquitous chronic inflammatory condition affecting the supporting structures of the teeth, initiated by dysbiotic bacterial biofilms. It is now recognized not merely as a localized oral infection but as a significant source of low-grade systemic inflammation [2]. The potential for periodontal pathogens and inflammatory mediators to breach systemic circulation and compromise the blood–brain barrier (BBB) has led to the hypothesis that periodontitis may be a modifiable risk factor for neurodegenerative conditions, including Alzheimer’s disease (AD) [1,3].

Clarification of the exposure construct. In this review, periodontitis is conceptualized primarily as a chronic, host-mediated inflammatory burden rather than as a localized intraoral infection or as a single ordinal severity category. Tissue destruction in periodontitis results predominantly from a dysregulated host inflammatory and immune response to a dysbiotic subgingival biofilm, and it is this sustained inflammatory load, rather than the presence of particular microorganisms alone, that provides the biologically plausible link to neuroinflammation [2,3]. Accordingly, the periodontal measures reported by the included studies, i.e., probing depth (PD/PPD), clinical attachment loss (CAL), bleeding on probing (BOP), radiographic bone loss (MBL/RABL), and composite measures such as the periodontal inflamed surface area (PISA), are interpreted here as alternative operationalizations of the same underlying construct, namely the magnitude of the host periodontal inflammatory burden. Where studies applied severity thresholds (for example, CDC/AAP case definitions, European Workshop in Periodontology criteria, or radiographic bone-loss cut-offs), these are treated as study-specific operationalizations of that burden rather than as interchangeable severity grades. Because the included studies span different periodontal classification eras and use heterogeneous case definitions, the comparability of severity grading across studies is addressed explicitly as a methodological issue in the interpretation of our findings.

Furthermore, epidemiological evidence has begun to substantiate these biological theories. Recent studies suggest that individuals with chronic periodontitis may exhibit a higher risk of cognitive decline compared to periodontally healthy individuals [4,5]. This association appears particularly robust in older adults, where the cumulative effect of long-standing inflammation may accelerate neurodegenerative processes [6]. Given the projected increase in dementia cases worldwide, identifying and managing modifiable risk factors such as periodontal disease could have profound implications for public health strategies aimed at preserving cognitive function in aging populations [7].

To accurately assess this spectrum, standardized neuropsychological instruments are essential. Commonly utilized tools include the Mini-Mental State Examination (MMSE) for global cognition screening, the Montreal Cognitive Assessment (MoCA) which is more sensitive to mild impairment, Addenbrooke’s Cognitive Examination (ACE) for comprehensive multi-domain evaluation, and the Alzheimer’s Disease Assessment Scale–Cognitive Subscale (ADAS-Cog) for monitoring disease progression. AD, the most common cause of dementia, is pathologically characterized by extracellular amyloid-beta (Aβ) plaques and intracellular neurofibrillary tangles composed of hyperphosphorylated tau protein. The amyloid cascade hypothesis posits that abnormal processing of amyloid precursor protein (APP) leads to synaptic injury, neuronal dysfunction, and cell death. Recent research has increasingly focused on modifiable risk factors that may influence this pathological cascade, with periodontal disease emerging as a significant potential contributor.

**Figure 1:**
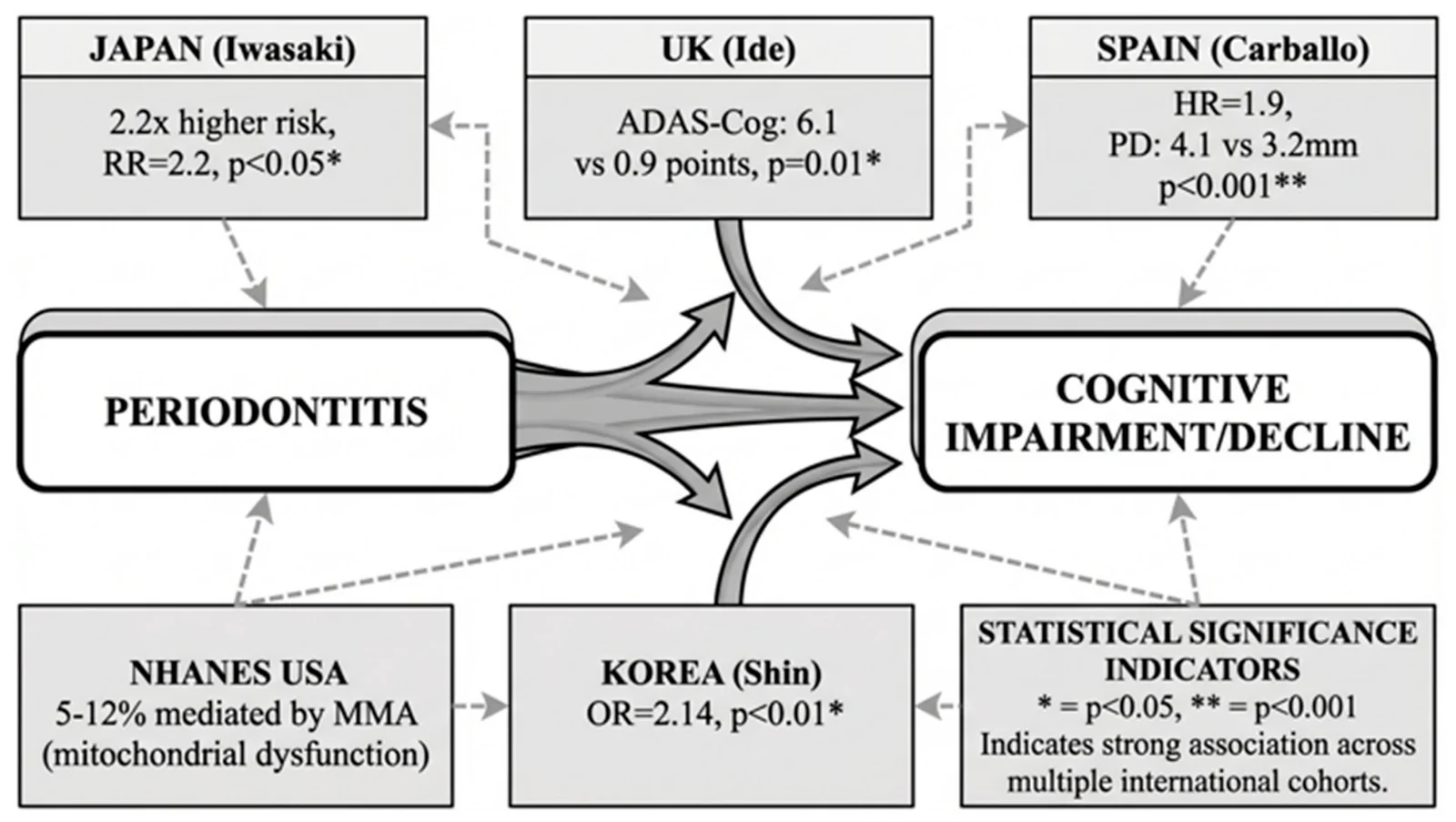
Diagrammatic illustration of key international studies linking periodontitis to cognitive impairment outcomes.

A growing body of evidence from cross-sectional and prospective cohort studies indicates an association between periodontitis and cognitive status. Studies from diverse geographical regions including Japan, the UK, and the USA have linked severe periodontitis to lower cognitive scores and an increased risk of decline (Figure 1). Specifically, severe periodontitis has been associated with a higher risk of incident MCI and accelerated progression in established AD patients.

**Figure 2:**
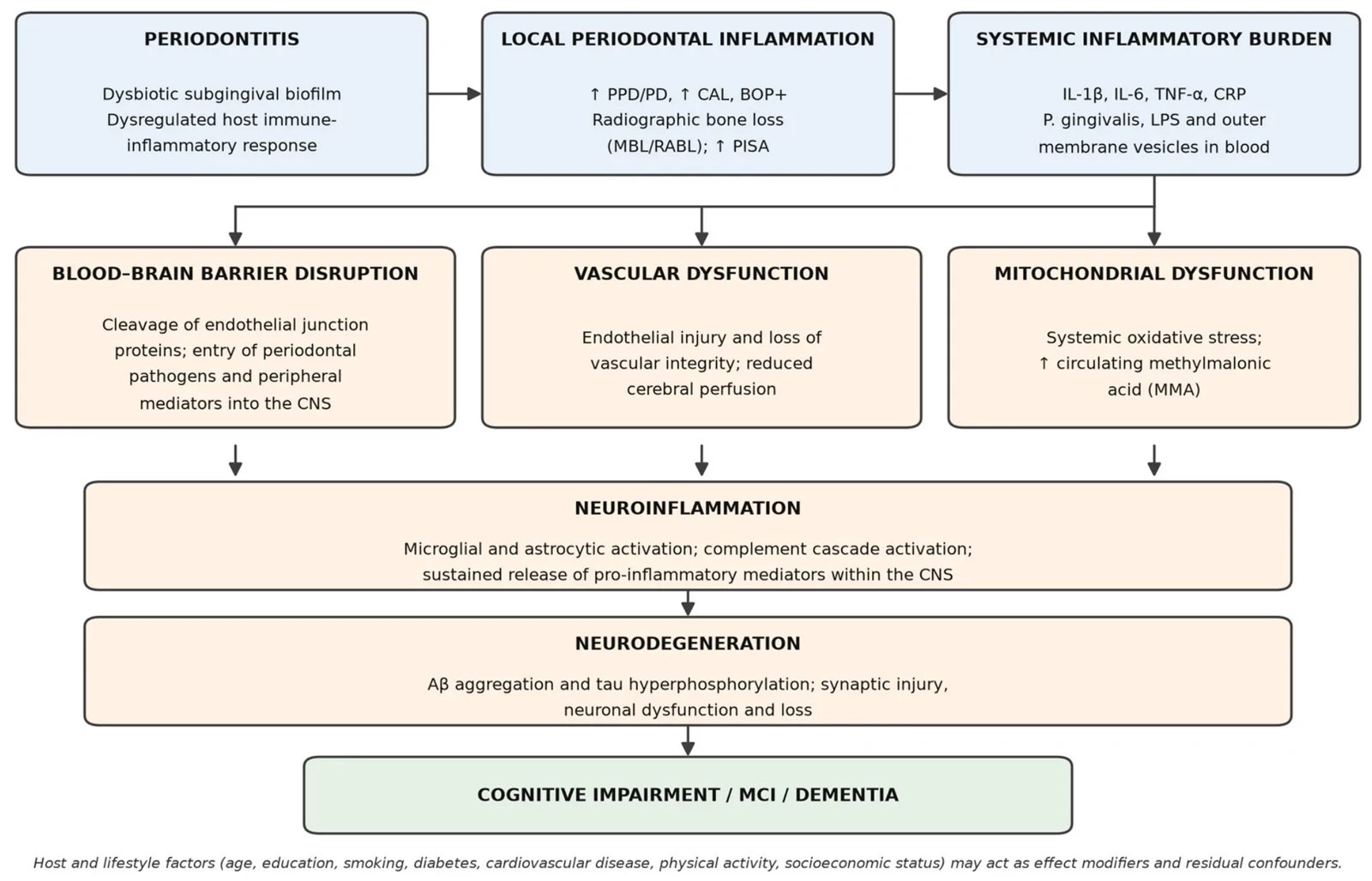
Schematic overview of the proposed oral-systemic pathway linking periodontitis, systemic inflammation, neuroinflammation and cognitive impairment. The exposure is depicted as a host-mediated periodontal inflammatory burden that propagates through blood–brain barrier disruption, vascular injury and mitochondrial dysfunction, converging on neuroinflammation and neurodegeneration.

Several biological mechanisms have been proposed to explain this association and are summarized in Figure 2. They can be grouped into direct and indirect pathways:

Added value of the present synthesis: The epidemiological association between periodontal disease and cognitive impairment has already been addressed in previous systematic reviews and meta-analyses [8,9,10,11]. The present synthesis is therefore not positioned as re-establishing that association. Its contribution is to consolidate the epidemiological context of the association and to organize the candidate biological pathways still incompletely resolved through which a host-mediated periodontal inflammatory burden may plausibly contribute to cognitive impairment, including the more recent cohort and Alzheimer’s disease data published through 2025 (Figure 2).

Direct Pathological Pathways: Encapsulated Porphyromonas gingivalis (serotypes K1/K2) induces memory deficits, increases alveolar bone resorption, and elevates hippocampal Aβ1-42 and tau hyperphosphorylation in animal models [12]. Systemic Inflammation: Periodontal disease induces a chronic systemic inflammatory state, characterized by elevated levels of pro-inflammatory cytokines (IL-1β, IL-6, TNF-α). These mediators can cross the BBB, activating microglia and astrocytes, thereby driving neuroinflammation [2,13,14,15]. Furthermore, Porphyromonas gingivalis, its outer membrane vesicles (OMVs), and lipopolysaccharides (LPSs) may increase BBB permeability, facilitating the entry of pathogens and peripheral inflammatory mediators into the central nervous system (CNS) [16,17]. Mitochondrial Dysfunction: Periodontal inflammation contributes to systemic oxidative stress, which may lead to mitochondrial dysfunction. Circulating methylmalonic acid (MMA), a marker of mitochondrial dysfunction, has been shown to partially mediate the association between periodontitis and cognitive decline [7]. Vascular Mechanisms: Periodontal pathogens and their OMVs can cleave endothelial junction proteins, compromising vascular integrity and potentially affecting cerebral perfusion [18]. Complement Activation: Periodontal infection has been shown to activate the complement cascade, thereby contributing to sustained neuroinflammatory responses and neurodegeneration in animal models of AD. Experimental studies suggest that periodontal pathogens, particularly Porphyromonas gingivalis and its virulence factors such as LPSs, may stimulate microglial activation and upregulate complement-related inflammatory mediators within the CNS [19]. Clinical Signals and Epidemiology: Long-standing periodontitis has been associated with approximately a twofold increased risk of AD, suggesting that chronic periodontal inflammation may contribute to neurodegenerative processes [18]. In addition, reducing the oral load of Porphyromonas gingivalis has been reported to be associated with slower cognitive decline in a clinical subanalysis [20].

Despite the growing body of literature linking oral health to systemic well-being, a comprehensive synthesis of how specific periodontal conditions influence cognitive health is necessary to clarify these complex pathways. This systematic review and meta-analysis aim to synthesize and quantitatively analyze the clinical evidence regarding the association between periodontal disease and cognitive impairment, examining both the strength of the epidemiological data and the biological plausibility of the proposed mechanisms. Specifically, this study seeks to summarize the periodontal parameters assessed in the literature such as Probing Pocket Depth (PPD), Clinical Attachment Loss (CAL), and Marginal Bone Loss (MBL) while reviewing the cognitive assessment tools utilized, including the MMSE, MoCA, and ADAS-Cog. Furthermore, we aim to provide a pooled quantification of the associations between periodontal disease severity and cognitive outcomes, evaluate the risk-of-bias profile of the included studies, and determine the overall strength of the current evidence.

### Aim of the Study

The primary aim of this study was to systematically review and conduct a meta-analysis of the clinical evidence regarding the association between periodontal disease and cognitive impairment in adult populations. Specifically, the study sought to:

- Summarize the periodontal parameters assessed in the literature (e.g., Probing Pocket Depth, Clinical Attachment Loss, Marginal Bone Loss).
- Review the cognitive assessment tools and trajectories used (e.g., MMSE, MoCA, ADAS-Cog).
- Quantify the associations between periodontal disease severity and cognitive outcomes through meta-analytical methods.
- Synthesize the risk-of-bias profile of included studies and evaluate the underlying biological plausibility.

## 2. Materials and Methods

### 2.1. Study Design and Registration

This systematic review was conducted in accordance with the Preferred Reporting Items for Systematic Reviews and Meta-Analyses (PRISMA) 2020 statement [21] File S1. The protocol was registered in the International Prospective Register of Systematic Reviews (PROSPERO) under registration number CRD420251267024. The completed PRISMA 2020 checklist is provided as Supplementary File S1.

### 2.2. PECO Question and Eligibility Criteria

The focused question was: “Is periodontal disease associated with an increased risk or progression of cognitive impairment in adults?”

Eligibility criteria (PECO) were defined as follows:

- Population: Adults (≥18 years), focusing on general older adult populations and cohorts with Alzheimer’s disease.
- Exposure: Periodontal disease conceptualized as a host-mediated chronic inflammatory burden [2,3] and operationalized in the included studies by clinical parameters (PPD, CAL, BOP), radiographic bone loss (MBL/RABL), or validated composite indices and case definitions (e.g., CDC/AAP criteria, EWP criteria, PISA). No minimum severity threshold was applied at the eligibility stage; studies reporting periodontitis relative to a healthier comparator at any degree of severity were eligible.
- Comparator: Individuals with healthy periodontium, gingivitis only, or lower severity of periodontal disease.
- Outcome: Cognitive impairment assessed via validated tools measuring incidence, progression, or rate of decline.
- Study Design: Human observational studies including prospective and retrospective cohorts, clinical trials, and cross-sectional studies published in English.

### 2.3. Search Strategy

A systematic electronic search was performed in PubMed, Web of Science, and Scopus databases for studies published between 1 January 2015 and 5 December 2025; the searches were last executed in December 2025. Search concepts combined terms for the exposure and for the outcome: (periodontitis OR periodontal disease OR periodontal inflammation) AND (cognition OR cognitive impairment OR cognitive decline OR MCI OR dementia OR Alzheimer) AND (cohort OR longitudinal OR prospective OR case-control OR cross-sectional). The complete database-specific search strategies, including field tags, controlled vocabulary and any limits applied, are provided in the Supplementary Materials (Table S1). No language filter was applied within the searches themselves; the English-language restriction was applied at the eligibility stage.

### 2.4. Study Selection and Data Extraction

Screening was performed independently by two reviewers, first by title and abstract, followed by full-text review. Disagreements were resolved by consensus. Data extraction included study design, participant demographics, sample size, periodontal metrics, and cognitive assessment tools. A qualitative synthesis of findings was performed.

### 2.5. Risk of Bias Assessment

Methodological quality was assessed using the Newcastle–Ottawa Scale (NOS) for non-randomized observational studies. Studies were evaluated across three domains: Selection (representativeness), Comparability (control for confounders), and Outcome (assessment method and follow-up). Studies were classified as having Low, Moderate, or High risk of bias.

### 2.6. Data Synthesis and Statistical Analysis

Studies that reported a comparable quantitative estimate of the association between periodontitis and cognitive impairment were eligible for quantitative synthesis. Effect estimates were extracted as hazard ratios (HRs), odds ratios (ORs) or risk ratios (RRs) together with their 95% confidence intervals (CIs) and were analyzed on the natural logarithmic scale, treating the different measures as generic relative risks. Because contributing studies reported conceptually analogous measures of the same association rather than one common effect measure, the synthesis yields an overall summary association estimate rather than a measure-specific pooled risk, and this limitation is carried explicitly through the interpretation of the results.

Pooling was performed with inverse-variance weighting. A random-effects model using the DerSimonian–Laird estimator was prespecified as the primary analysis, and a fixed-effect model was fitted for comparison. Between-study heterogeneity was quantified with Cochran’s Q statistic, the I^2^ statistic and the between-study variance (τ^2^). Two sensitivity analyses were prespecified: (i) restriction to prospective cohort studies only, and (ii) substitution of an alternative periodontitis case definition (EWP criteria instead of CDC/AAP criteria) for the study that reported both. Statistical significance was set at a two-sided *p* value below 0.05. Because fewer than ten studies contributed to the synthesis, formal assessment of publication bias was not performed.

The certainty of the body of evidence was judged qualitatively, on the basis of the observational design of the included studies, their methodological limitations, heterogeneity in exposure and outcome definitions, and the potential for residual confounding and reporting bias. This judgment is a qualitative interpretation and is not presented as a formal GRADE assessment.

## 3. Results

### 3.1. Study Selection

**Figure 3:**
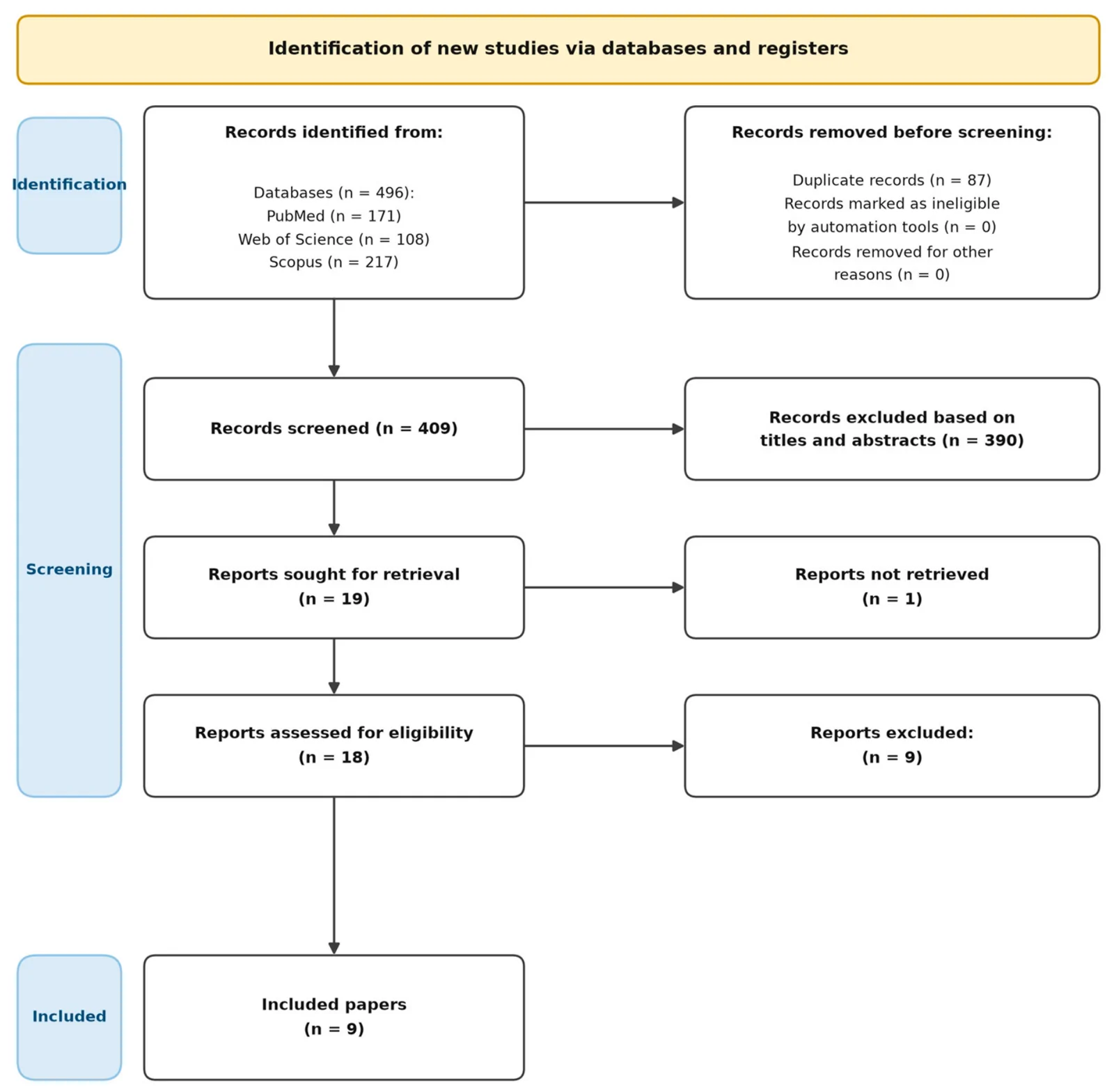
PRISMA flow diagram of study selection process.

The systematic search identified 496 records (PubMed, 171; Web of Science, 108; Scopus, 217). After 87 duplicate records were removed, 409 records were screened, of which 390 were excluded on the basis of title and abstract; the full texts of 19 reports were therefore sought for retrieval. One report could not be retrieved, leaving 18 reports assessed for eligibility. Nine reports were excluded after full-text assessment, and nine studies [22,23,24,25,26,27,28,29,30] met the inclusion criteria and were included in the final qualitative synthesis (Figure 3). The included studies were published between 2016 and 2025, originating from diverse geographic locations including Europe (Spain, Sweden, UK), Asia (Japan, Korea, Taiwan, Turkey), and South America (Colombia).

### 3.2. Study Characteristics

The included studies comprised prospective cohort designs, cross-sectional analyses, and observational studies in specific patient populations. Sample sizes ranged from 30 to 775 participants. Populations included community-dwelling older adults and specific cohorts of patients with AD. A summary of the studies included is presented in Table 1.

### 3.3. Periodontal Parameters’ Findings

The studies utilized various definitions for periodontal disease, including clinical parameters (Probing Depth [PD], Clinical Attachment Loss [CAL], Bleeding on Probing [BOP]) and radiographic measures (Marginal Bone Loss [MBL]). As detailed in Table 2, Carballo et al. [27] found significant differences in PD and AL between periodontitis and non-periodontitis groups, while Arévalo-Caro et al. [30] identified gingival erythema as a strong predictor of MCI. Further analysis of periodontal parameters highlights that severe periodontitis, defined by CDC/AAP criteria or radiographic bone loss, was significantly associated with cognitive outcomes in longitudinal cohorts [23] and cross-sectional studies [24]. In AD cohorts, disease progression correlated with periodontal inflammation. Karaduran et al. [29] found significantly higher BOP and CAL in late-stage AD patients, while Ide et al. [22] reported that periodontitis was common even in mild-to-moderate AD cases.

A summary of the main periodontal findings across all included studies is provided in Table 3; across studies, greater periodontal severity whether measured by probing depth, clinical attachment loss, or radiographic bone loss was consistently associated with poorer cognitive outcomes.

### 3.4. Cognitive Outcomes and Associations

Cognitive outcomes were assessed using standardized tools such as MMSE, MoCA, and ADAS-Cog. As shown in Table 4, findings from Carballo et al. [27], Chen et al. [28], and Arévalo-Caro et al. [30] indicate that specific oral health conditions and inflammatory signs predict cognitive decline and MCI status. In addition, data from Nilsson et al. [25] and Iwasaki et al. [23,26] indicate that severe periodontitis is associated with incident MCI (OR = 3.58) and with future cognitive decline (RR = 2.2) over longitudinal follow-up. Finally, findings from Shin et al. and the AD cohorts [22,24,29] demonstrated a consistent association between periodontitis history and cognitive impairment, reinforcing the potential role of periodontal disease in accelerating AD progression.

### 3.5. Risk of Bias Assessment Results

**Figure 4:**
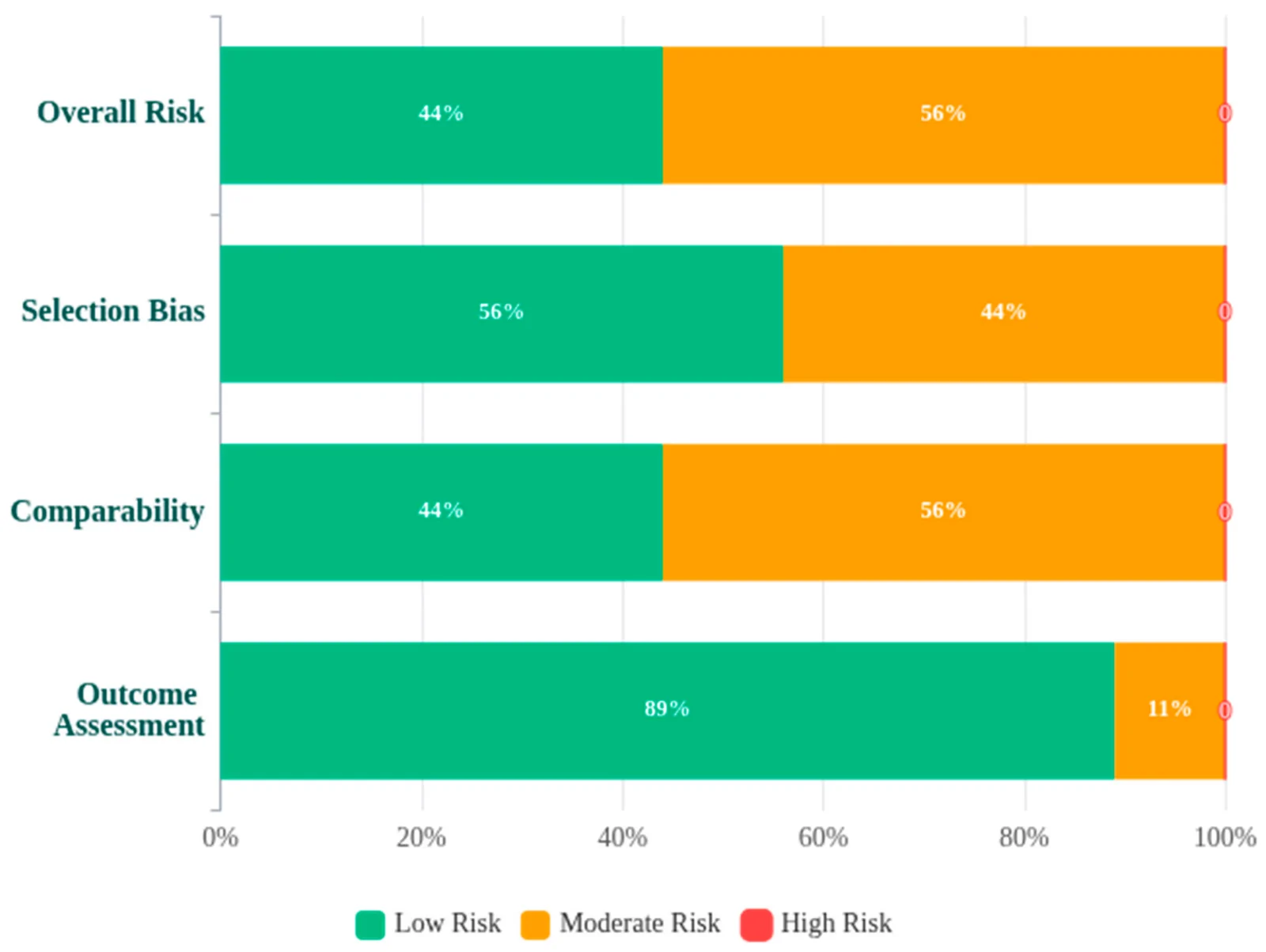
Summary risk-of-bias assessment of included studies using the Newcastle–Ottawa Scale.

Assessment using the Newcastle–Ottawa Scale indicated that three studies were at low overall risk of bias and six at moderate overall risk of bias (Table 5). No study was classified as having a high risk of bias. A visual summary of the risk-of-bias assessment is provided in Figure 4.

### 3.6. Meta-Analysis Results

A meta-analysis was conducted to quantify the association between periodontal disease and cognitive impairment across the included studies. Four studies including Carballo et al. [27], Iwasaki et al. [23,26], and Shin et al. [24] met the criteria for quantitative synthesis, encompassing a total of 554 participants across cohorts from Spain, Japan, and South Korea.

#### 3.6.1. Pooled Effect Estimate

Using a random-effects model (DerSimonian–Laird method), the pooled estimate indicated an approximately twofold overall association estimate between periodontitis and cognitive impairment (pooled effect = 2.12; 95% CI: 1.51–2.98; *p* < 0.001). It should be noted that the pooled effect estimate combines different effect measures specifically hazard ratios [23,27], an odds ratio [26], and a risk ratio [24] treated as generic relative risks for the purposes of quantitative synthesis. This approach is commonly used when studies report conceptually analogous measures but introduces a degree of imprecision; in particular, odds ratios may overestimate the true relative risk when outcome prevalence is not rare, meaning the pooled estimate of 2.12 should be interpreted as an approximate summary measure rather than a precise effect size.

**Figure 5:**
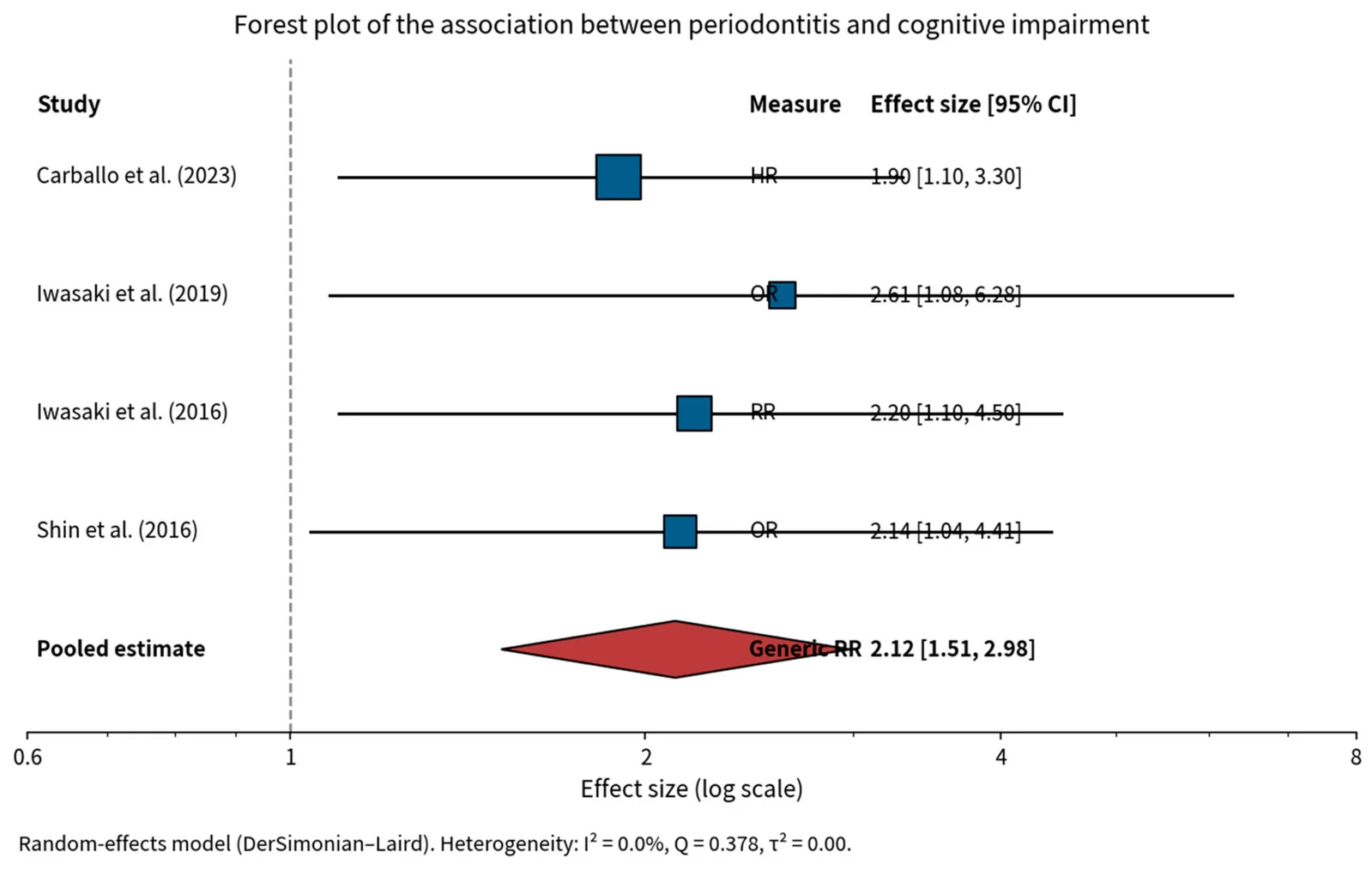
Forest plot of the association between periodontitis and cognitive impairment [23,24,26,27].

The study-specific estimates were HR 1.90 (95% CI: 1.10–3.30) for Carballo et al. [27], OR 2.61 (95% CI: 1.08–6.28) for Iwasaki et al. [26], RR 2.20 (95% CI: 1.10–4.50) for Iwasaki et al. [23], and OR 2.14 (95% CI: 1.04–4.41) for Shin et al. [24]. These results are summarized in Table 6 and displayed in the forest plot in Figure 5. Notably, all four individual study estimates exceeded 1.0 with confidence intervals excluding the null, indicating that each study independently reached statistical significance.

#### 3.6.2. Heterogeneity Assessment

No statistical heterogeneity was observed across the included studies (I^2^ = 0.0%; Cochran’s Q = 0.378; df = 3). The between-study variance (τ^2^) was 0.00, indicating that the variability in effect estimates was attributable entirely to within-study sampling error rather than true between-study differences. As a result, the fixed-effect and random-effects models produced identical pooled estimates (2.12; 95% CI: 1.51–2.98). The absence of heterogeneity suggests remarkable consistency in the direction and magnitude of the association across studies, despite differences in study design, population, and follow-up duration.

#### 3.6.3. Sensitivity Analyses

Two sensitivity analyses were conducted to assess the robustness of the primary findings. First, restricting the analysis to prospective cohort studies only (k = 3; excluding the cross-sectional study by Shin et al. [24] yielded a nearly identical pooled estimate (2.11; 95% CI: 1.43–3.12; I^2^ = 0.0%), confirming that the inclusion of the cross-sectional study did not materially influence the overall result. Second, substituting the alternative periodontitis definition (EWP instead of CDC/AAP) for Iwasaki et al. [26] produced a marginally higher pooled estimate (2.21; 95% CI: 1.57–3.12; I^2^ = 0.0%), indicating that the association was not sensitive to the choice of periodontitis case definition.

#### 3.6.4. Methodological Considerations

Several methodological caveats should be acknowledged when interpreting these findings. First, the pooled estimate combines different effect measures hazard ratios (HRs), odds ratios (ORs), and risk ratios (RRs) as generic relative risks. While this approach is common when studies report different but conceptually analogous measures, it introduces a degree of imprecision, particularly because ORs tend to overestimate the relative risk when the outcome is not rare. Second, the included studies varied in design (one cross-sectional, three prospective cohort), geographic setting (Spain, Japan, South Korea), follow-up duration (cross-sectional to 5 years), and periodontitis definitions (CDC/AAP classification, radiographic bone loss criteria), although all studies applied established diagnostic criteria. Third, with only four studies included in the meta-analysis, the statistical power to detect heterogeneity is limited, and the I^2^ of 0% should be interpreted with caution; it may reflect insufficient power to detect true between-study variation rather than genuine homogeneity. Similarly, formal assessment of publication bias (e.g., funnel plot, Egger’s test) was not feasible due to the small number of studies.

#### 3.6.5. Summary

Across four studies, periodontitis was consistently and significantly associated with an approximately twofold overall association estimate for cognitive impairment (pooled effect estimate = 2.12; 95% CI: 1.51–2.98; I^2^ = 0.0%). This association was robust across sensitivity analyses removing the cross-sectional study and using alternative periodontitis definitions. While the consistency of findings across geographically and methodologically diverse studies strengthens confidence in the association, the small number of included studies, the combination of different effect measures, and the inability to assess publication bias warrant cautious interpretation. These findings support a meaningful association between periodontal disease and cognitive impairment, although causality cannot be established from observational data alone. Further large-scale prospective studies with standardized periodontal and cognitive outcome definitions are needed to confirm and extend these findings.

## 4. Discussion

The aim of this systematic review was to evaluate the clinical evidence linking periodontal disease with cognitive impairment in adults and to determine whether the association is consistent across periodontal measures, cognitive outcomes, and study designs. Overall, the review found a coherent pattern: greater periodontal disease severity was associated with poorer cognitive performance, increased risk of incident mild cognitive impairment, and faster cognitive decline. This pattern was seen in community-based older adult cohorts as well as in patients with established AD, where active periodontal inflammation was associated with more rapid deterioration. Taken together, the findings support an association that is epidemiologically consistent and biologically plausible, although not yet sufficient to confirm causality.

These findings are broadly consistent with prior systematic reviews and meta-analyses. Nadim et al. [8] reported that periodontal disease was associated with a higher risk of dementia, with pooled relative risks of 1.38 overall and 1.18 in cohort studies. Asher et al. [9] similarly found that poor periodontal health and tooth loss were associated with both cognitive decline (OR 1.23, 95% CI 1.05–1.44) and dementia (HR 1.21, 95% CI 1.07–1.38), although they judged the overall evidence to be low and methodologically heterogeneous. Said-Sadier et al. [10] also concluded that all included studies showed evidence of an association between periodontitis and cognitive impairment or dementia, but they did not perform a meta-analysis because of heterogeneity. More recently, Dibello et al. [11] found significant associations between periodontal disease and cognitive disorders (RR 1.25, 95% CI 1.11–1.40), cognitive decline in longitudinal studies (RR 3.01, 95% CI 1.52–5.95), and dementia (RR 1.22, 95% CI 1.10–1.36). The present review therefore does not overturn prior conclusions; rather, it reinforces them and extends them by incorporating newer studies through 2025, including recent cohort data, updated biomarker-linked findings, and additional evidence from AD populations that better illustrate disease progression and inflammatory burden [8,9,10,11]. Because the epidemiological association has already been addressed by these earlier syntheses, the added value of the present review lies less in restating it than in defining its epidemiological context and in organizing the candidate biological pathways still incompletely resolved through which a host-mediated periodontal inflammatory burden may plausibly contribute to cognitive decline (Figure 2). Interpretation of the exposure across studies nevertheless requires caution: because the included studies span different periodontal classification eras and applied heterogeneous case definitions, the severity thresholds used are not directly interchangeable, and comparisons of effect magnitude across severity strata should be regarded as approximate.

The risk-of-bias findings should also be considered when interpreting the results. Using the Newcastle–Ottawa Scale, three included studies were judged to have low overall risk of bias and six had moderate risk of bias; none was classified as high risk. This supports the internal consistency of the literature, but it does not eliminate key limitations. Most studies were observational, several were cross-sectional, and comparability domains were commonly limited by incomplete adjustment for important confounders. Accordingly, a formal GRADE assessment was not performed, and the statement that the overall certainty of the evidence is low should be read as a qualitative interpretation based on the observational design of the included studies and their methodological limitations, rather than as a formal GRADE rating. The certainty was downgraded by observational design, heterogeneity in exposure and outcome definitions, residual confounding, and the absence of direct interventional evidence showing that periodontal treatment prevents cognitive decline.

From a clinical perspective, the current evidence justifies greater awareness of oral health as a potentially relevant factor in cognitive aging and supports risk communication between dental and medical teams. However, the evidence does not yet justify formal population-level cognitive screening programs based solely on periodontal status. The more defensible implication at present is that periodontal assessment and treatment may form part of comprehensive care for older adults, particularly those at elevated risk for cognitive decline, as effective management of periodontitis may represent a strategic intervention to help slow cognitive deterioration, especially in patients with AD [22,29,31]. If future interventional studies show that periodontal therapy changes cognitive trajectories, the implications would be substantial for prevention strategies, multidisciplinary geriatric care, and mechanistic understanding of inflammation-related neurodegeneration.

Several limitations should be acknowledged when interpreting these findings. First, publication bias cannot be excluded because no formal funnel-plot assessment was possible and grey literature searching was not a major component of the review process. Second, of the nine included studies, only four provided sufficiently comparable data to permit quantitative synthesis. While pooled effect estimates were generated, considerable heterogeneity among studies in terms of periodontal case definitions, cognitive evaluation methods, and study methodologies limited the comprehensiveness of the meta-analysis and warrants cautious interpretation of the findings. Third, residual confounding remains a major concern in this literature, particularly for smoking, diabetes, cardiovascular disease, education, physical activity, and socioeconomic status, all of which may influence both periodontal status and cognition. Future studies should therefore use standardized periodontal and neurocognitive definitions, stronger confounder control, longer follow-up, and ideally randomized periodontal intervention designs linked to biomarker and clinical endpoints.

## 5. Conclusions

This systematic review provides consistent evidence of an approximately twofold overall association between periodontal disease, understood as a host-mediated inflammatory burden, and cognitive impairment, including an increased risk of mild cognitive impairment and faster decline in some AD cohorts. However, because the available evidence is predominantly observational and overall certainty is low, the findings should be interpreted as supportive rather than definitive. Optimizing periodontal health remains a reasonable component of healthy aging strategies, while stronger longitudinal and interventional studies are needed to determine whether periodontal treatment can meaningfully alter cognitive outcomes.

## Figures and Tables

**Table 1:** Characteristics of Included Studies (Summary of Included Studies: 2016–2025).

Study (Year)	Country	Design	Population & Setting	Sample (N)	Aim of the Study
Ide et al. (2016) [22]	UK	Observational (AD)	Non-smoking community-dwelling AD patients	59	Determine if periodontitis in AD is associated with increased dementia severity and cognitive decline
Iwasaki et al. (2016) [23]	Japan	Prospective Cohort	Community-dwelling adults (mean 79.3 years)	85	Examine longitudinal relationship of severe periodontitis with cognitive decline
Shin et al. (2016) [24]	Korea	Cross-sectional	Dentate elderly 60–86 years from Yangpyeong Cohort	189	Investigate association between periodontitis and cognitive impairment in elderly Koreans
Nilsson et al. (2018) [25]	Sweden	Cross-sectional	Adults 60–99 years from SNAC cohort	775	Evaluate association between periodontitis, tooth loss, and cognitive functions
Iwasaki et al. (2019) [26]	Japan	Prospective Cohort	Dentate community-dwelling adults (≥75 years)	179	Determine whether periodontitis and inflammation are associated with incident MCI (5-year follow-up)
Carballo et al. (2023) [27]	Spain	Prospective Cohort	Elderly hypertensive adults (≥60 years)	101	Assess association of periodontitis with cognitive decline and blood-based AD biomarkers
Chen et al. (2024) [28]	Taiwan	Prospective Cohort	Non-demented community of older adults (≥65 years)	516	Investigate relationships between baseline oral conditions and cognition (6-year follow-up)
Karaduran et al. (2024) [29]	Turkey	Prospective (AD)	Alzheimer’s patients (50–89 years)	90	Investigate effect of periodontitis and occlusal relationship on progression rate of AD
Arévalo-Caro et al. (2025) [30]	Colombia	Prospective Cohort	Elderly adults (≥65 years) from Bogotá	30	Identify specific periodontal indices associated with MCI as predictive markers

Abbreviations: AD = Alzheimer’s Disease; MCI = Mild Cognitive Impairment.

**Table 2:** Periodontal parameters assessed in the included studies.

Study (Year)	Parameters	Key Quantitative Findings (Mean ± SD or %)	Definitions/Notes
Ide et al. (2016) [22]	PD, BOP, Plaque	Mean PD: 2.5 ± 0.4 mm. BOP: 13.6%. Plaque presence: 89% of participants.	Focus on the systemic inflammation link in mild-to-moderate AD patients.
Iwasaki et al. (2016) [23]	CAL, PD	CDC/AAP Severe Criteria: CAL ≥ 2 sites ≥ 6 mm; PD ≥ 1 site ≥ 5 mm.	Mean mm values not reported. Focus on categorical definition.
Shin et al. (2016) [24]	RABL	Moderate: ≥2 sites RABL ≥ 4 mm; Severe: ≥2 sites RABL ≥ 6 mm	Measured from CEJ to alveolar crest. Clinical indices (PD/CAL) not reported.
Nilsson et al. (2018) [25]	MBL, PD	MBL ≥ 4 mm at ≥30% of sites found in 115/585 participants. PD ≥ 5 mm at ≥30% of teeth in 6.4%.	MBL used as main severity indicator. Pockets ≥ 5 mm NOT significantly associated with cognition.
Iwasaki et al. (2019) [26]	PPD, AL, BOP	Severe vs. Not Severe: PPD: 3.5 ± 0.7 vs. 2.6 ± 0.6 mm (*p* < 0.01); AL: 4.8 ± 1.3 vs. 3.4 ± 0.8 mm (*p* < 0.01); BOP: 16.1% vs. 10.6% (*p* = 0.01)	PISA also analyzed. MBL, GI, and PI not measured.
Carballo et al. (2023) [27]	PD, AL	PD: Periodontitis 3.7 ± 1.0 mm vs. Non-Perio 2.8 ± 0.8 mm (*p* < 0.001); AL: Periodontitis 4.1 ± 1.1 mm vs. Non-Perio 3.2 ± 1.2 mm (*p* = 0.001)	CAL at >2 non-adjacent teeth used for definition. MBL, GI, and PI not measured.
Chen et al. (2024) [28]	Periodontal Status (CAL)	Not reported in original study (Classified categorically)	Classified as: Health, Gingivitis, or Periodontitis.
Karaduran et al. (2024) [29]	CAL, PPD, BOP	CAL by AD Stage: I (4.70 mm), II (6.30 mm), III (6.49 mm); PPD by AD Stage: I (3.90 mm), II (4.20 mm), III (4.70 mm); BOP significantly higher in Stage III AD.	Data stratified by AD stage (CDR). PI recorded but values not specified.
Arévalo-Caro et al. (2025) [30]	PD, Gingival Erythema, PI	PD (Freq ≥ 4 mm): MCI 0.10 vs. Control 0.03 (*p* = 0.015); Gingival Erythema: MCI 0.39 vs. Control 0.0 (*p* = 0.003)	All severe periodontitis cases were found in the MCI group. Gingival erythema was a strong predictor.

Abbreviations: PPD = Probing Pocket Depth; PD = Probing Depth; AL/CAL = Clinical Attachment Level/Loss; BOP = Bleeding on Probing; MBL = Marginal Bone Loss; RABL = Radiographic Alveolar Bone Loss; AD = Alzheimer’s Disease; GI = Gingival Index; PI = Plaque Index; MCI = Mild Cognitive Impairment; CEJ = Cementoenamel Junction; CDC = Centers for Disease Control and Prevention; AAP = American Academy of Periodontology; CDR = Clinical Dementia Rating.

**Table 3:** Summary of the main periodontal parameters’ findings.

Study	Parameter	Key Findings	Significance/Notes
Ide et al. (2016) [22]	PD, BOP	Presence of periodontitis	6-fold increase in cognitive decline rate
Shin et al. (2016) [24]	RABL	Moderate/Severe bone loss	History of periodontitis independently associated with CI
Nilsson et al. (2018) [25]	MBL	MBL ≥ 4 mm at ≥30% of sites	Associated with lower MMSE scores (OR = 2.7)
Iwasaki et al. (2019) [26]	PPD, CAL, BOP	Severe periodontitis associated with higher PPD/CAL	Associated with incident MCI
Carballo et al. (2023) [27]	PPD, AL	PPD: 3.7 mm vs. 2.8 mm	Significant difference (*p* < 0.001)
Karaduran et al. (2024) [29]	CAL, PPD, BOP	Stage III AD had higher CAL/PPD	BOP correlated with cognitive decline

Abbreviations: PPD = Probing Pocket Depth; AL/CAL = Clinical Attachment Level/Loss; BOP = Bleeding on Probing; MBL = Marginal Bone Loss; RABL = Radiographic Alveolar Bone Loss; AD = Alzheimer’s Disease.

**Table 4:** Cognitive Tools and Association Outcomes.

Study (Year)	Cognitive Tool(s)	Association Outcome (Effect Size, *p*-Value)	Summary Conclusion
Ide et al. (2016) [22]	ADAS-Cog, sMMSE	6-fold increase in decline rate. ADAS-Cog change 6.1 vs. 0.9 (*p* = 0.01).	Marked increase in decline in AD patients, possibly mediated by inflammation.
Iwasaki et al. (2016) [23]	MMSE (Decline ≥ 3)	Severe periodontitis linked to 2.2× risk of cognitive decline (RR = 2.2).	Severe periodontitis significantly associated with future cognitive decline in elderly Japanese.
Shin et al. (2016) [24]	MMSE-KC	History of periodontitis associated with CI (OR = 2.14). Interactions with smoking/exercise.	Independent association found; lifestyle factors such as smoking and exercise may modify risk for cognitive impairment.
Nilsson et al. (2018) [25]	MMSE, Clock Test	MBL ≥ 4 mm associated with MMSE < 25 (OR = 2.7, *p* = 0.013). Tooth loss OR = 2.0.	Cumulative history (bone loss) predicts impairment better than current pockets here.
Iwasaki et al. (2019) [26]	MMSE, Neurologist	Severe periodontitis associated with incident MCI (OR = 3.58). PISA score OR = 1.05.	Severe periodontitis is a significant risk factor for developing MCI over 5 years.
Carballo et al. (2023) [27]	ACE, MMSE	Lower MMSE (β = −1.5), increased progression risk (HR = 1.9). Elevated p-Tau & Aβ1-40.	Periodontitis linked to poor performance and faster decline; mediated by biomarkers.
Chen et al. (2024) [28]	MoCA-T, WMS-III	Caries linked to poor memory; incomplete dentition to poor attention. Perio status not significant for global MoCA-T.	Global MoCA-T score not significantly associated with periodontal status; specific oral conditions linked to specific cognitive domains
Karaduran et al. (2024) [29]	SMMT, CDR	Accelerated AD progression (ΔSMMT +0.425, *p* = 0.036). Correlated with BOP%.	Active inflammation accelerates cognitive decline in established AD.
Arévalo-Caro et al. (2025) [30]	MoCA	Gingival erythema (OR = 3.15, *p* = 0.003) and PD ≥ 4 mm (OR = 2.59) associated with MCI.	Inflammation and deep pockets are strong predictors for distinguishing MCI.

Abbreviations: ACE = Addenbrooke’s Cognitive Examination; MMSE = Mini-Mental State Examination; MMSE-KC = Korean Version of the Mini-Mental State Examination; p-Tau = Phosphorylated Tau; Aβ1-40 = Amyloid-Beta Peptide 1–40; AD = Alzheimer’s Disease; MoCA = Montreal Cognitive Assessment; MoCA-T = Montreal Cognitive Assessment—Taiwanese Version; WMS-III = Wechsler Memory Scale, Third Edition;; MCI = Mild Cognitive Impairment;; PISA = Periodontal Inflamed Surface Area;; RR = Relative Risk; SMMT = Standardized Mini-Mental Test; CDR = Clinical Dementia Rating; ADAS-Cog = Alzheimer’s Disease Assessment Scale–Cognitive Subscale; sMMSE = Standardized Mini-Mental State Examination.

**Table 5:** Risk of Bias Assessment: Detailed Domain Analysis (Newcastle–Ottawa Scale).

Study (Year)	Design	Selection	Comparability	Outcome	Overall
Ide et al. (2016) [22]	Observational (AD)	Mod	Low	Low	MODERATE
Iwasaki et al. (2016) [23]	Prospective Cohort	Mod	Low	Low	MODERATE
Shin et al. (2016) [24]	Cross-sectional	Mod	Mod	Low	MODERATE
Nilsson et al. (2018) [25]	Cross-sectional	Mod	Mod	Low	MODERATE
Iwasaki et al. (2019) [26]	Prospective Cohort	Low	Low	Low	LOW
Carballo et al. (2023) [27]	Prospective Cohort	Low	Low	Low	LOW
Chen et al. (2024) [28]	Prospective Cohort	Low	Low	Low	LOW
Karaduran et al. (2024) [29]	Prospective (AD)	Mod	Mod	Low	MODERATE
Arévalo-Caro et al. (2025) [30]	Prospective Cohort	Low	Mod	Low	MODERATE

Abbreviations: AD = Alzheimer’s Disease.

**Table 6:** Study-specific effect sizes included in the meta-analysis.

Study	Effect Measure	Effect Size	95% Confidence Interval
Iwasaki et al. (2016) [23]	RR	2.20	1.10–4.50
Shin et al. (2016) [24]	OR	2.14	1.04–4.41
Iwasaki et al. (2019) [26]	OR	2.61	1.08–6.28
Carballo et al. (2023) [27]	HR	1.90	1.10–3.30
Pooled estimate	Generic RR	2.12	1.51–2.98

Abbreviations: HR, hazard ratio; OR, odds ratio; RR, relative risk. Mixed-effect measures were synthesized as generic relative risks, consistent with the manuscript’s quantitative approach.

## Data Availability

All supporting data are available within the article and in the Supplementary Materials. Further data presented in this study are available on request from the corresponding author.

## Supplementary Materials

The following supporting information can be downloaded at: https://www.mdpi.com/article/10.3390/dj14100678/s1, Table S1. Complete search strategies as executed in each database); File S1: PRISMA 2020 checklist.

## Abbreviations

The following abbreviations are used in this manuscript:

AD	Alzheimer’s disease
ACE	Addenbrooke’s Cognitive Examination
ADAS-Cog	Alzheimer’s Disease Assessment Scale–Cognitive Subscale
Aβ	amyloid-beta
APP	amyloid precursor protein
BBB	blood–brain barrier
BOP	bleeding on probing
CAL	clinical attachment loss
CDC/AAP	Centers for Disease Control and Prevention/American Academy of Periodontology
CDR	Clinical Dementia Rating
CEJ	cementoenamel junction
CI	confidence interval
DSM-IV	Diagnostic and Statistical Manual of Mental Disorders, Fourth Edition
EWP	European Workshop in Periodontology
GI	gingival index
HR	hazard ratio
LPSs	lipopolysaccharides
MBL	marginal bone loss
MCI	mild cognitive impairment
MMSE	Mini-Mental State Examination
MMSE-KC	Korean version of the Mini-Mental State Examination
MoCA	Montreal Cognitive Assessment
MoCA-T	Montreal Cognitive Assessment—Taiwanese version
NOS	Newcastle–Ottawa Scale
OMV	outer membrane vesicle
OR	odds ratio
PD	probing depth
PECO	Population, Exposure, Comparator, Outcome
PI	plaque index
PISA	Periodontal Inflamed Surface Area
PPD	probing pocket depth
PRISMA	Preferred Reporting Items for Systematic Reviews and Meta-Analyses
PROSPERO	International Prospective Register of Systematic Reviews
RABL	radiographic alveolar bone loss
RR	relative risk
sMMSE	standardized Mini-Mental State Examination
SMMT	Standardized Mini-Mental Test
WMS-III	Wechsler Memory Scale, Third Edition
