# Supplementary material for: Association Between Periodontal Disease and Cognitive Impairment: A Systematic Review and Meta-Analysis
Source: Dent J (Basel). 2026 Oct 18;14(10):678. doi: 10.3390/dj14100678 (PMC13703410; doi:10.3390/dj14100678)
Supplement: Supplementary file 1 [file dentistry-14-00678-s001.zip › dentistry-4606580-Table S1.pdf]

Supplementary Material

Table S1. Complete search strategies as executed in each database

Database (interface)	Search strategy	Limits / date of last search	Records retrieved
PubMed (NLM)	("Periodontitis"[MeSH Terms] OR "Periodontal Diseases"[MeSH Terms] OR "Alveolar Bone Loss"[MeSH Terms] OR periodontitis[Title/Abstract] OR "periodontal disease"[Title/Abstract] OR "periodontal diseases"[Title/Abstract] OR "periodontal inflammation"[Title/Abstract]) AND ("Cognition Disorders"[MeSH Terms] OR "Cognitive Dysfunction"[MeSH Terms] OR "Dementia"[MeSH Terms] OR "Alzheimer Disease"[MeSH Terms] OR cognition[Title/Abstract] OR "cognitive impairment"[Title/Abstract] OR "cognitive decline"[Title/Abstract] OR "mild cognitive impairment"[Title/Abstract] OR MCI[Title/Abstract] OR dementia[Title/Abstract] OR Alzheimer*[Title/Abstract]) AND (cohort[Title/Abstract] OR longitudinal[Title/Abstract] OR prospective[Title/Abstract] OR "follow-up"[Title/Abstract] OR "case-control"[Title/Abstract] OR "cross-sectional"[Title/Abstract] OR observational[Title/Abstract])	Publication date 1 January 2015–5 December 2025; humans; no language limit applied in the search interface	171
Web of Science Core Collection (Clarivate)	TS=(periodontitis OR "periodontal disease*" OR "periodontal inflammation" OR "alveolar bone loss") AND TS=(cognition OR "cognitive impairment" OR "cognitive decline" OR "mild cognitive impairment" OR MCI OR dementia OR Alzheimer*) AND TS=(cohort OR longitudinal OR prospective OR follow-up OR "case-control" OR "cross-sectional" OR observational)	Timespan 2015–2025; no language limit applied in the search interface	108
Scopus (Elsevier)	TITLE-ABS-KEY(periodontitis OR "periodontal disease*" OR "periodontal inflammation" OR "alveolar bone loss") AND TITLE-ABS-KEY(cognition OR "cognitive impairment" OR "cognitive decline" OR "mild cognitive impairment" OR MCI OR dementia OR Alzheimer*) AND TITLE-ABS-KEY(cohort OR longitudinal OR prospective OR follow-up OR "case-control" OR "cross-sectional" OR	Publication years 2015–2025; no language limit applied in the search interface	217

Database (interface)	Search strategy	Limits / date of last search	Records retrieved
	observational) AND PUBYEAR > 2014 AND PUBYEAR < 2026		
